# Safety of routine early MRI in preterm infants

**DOI:** 10.1007/s00247-012-2426-y

**Published:** 2012-08-09

**Authors:** Annemarie Plaisier, Marlou M. A. Raets, Cynthia van der Starre, Monique Feijen-Roon, Paul Govaert, Maarten H. Lequin, Anneriet M. Heemskerk, Jeroen Dudink

**Affiliations:** 1 Division of Neonatology, Department of Pediatrics, Erasmus Medical Centre—Sophia, Dr. Molewaterplein, 60 3015 GJ Rotterdam, the Netherlands; 2 Division of Pediatric Radiology, Department of Radiology, Erasmus Medical Centre—Sophia, Rotterdam, the Netherlands; 3 Intensive Care, Department of Pediatrics and Pediatric Surgery, Erasmus Medical Centre—Sophia, Rotterdam, the Netherlands

**Keywords:** Premature infants, MRI, Safety management

## Abstract

**Background:**

Cerebral MRI performed on preterm infants at term-equivalent 30 weeks' gestational age (GA) is increasingly performed as part of standard clinical care.

**Objective:**

We evaluated safety of these early MRI procedures.

**Materials and methods:**

We retrospectively collected data on patient safety of preterm infants who underwent early MRI scans. Data were collected at fixed times before and after the MRI scan. MRI procedures were carried out according to a comprehensive guideline.

**Results:**

A total of 52 infants underwent an MRI scan at 30 weeks’ GA. Although no serious adverse events occurred and vital parameters remained stable during the procedure, minor adverse events were encountered in 26 infants (50%). The MRI was terminated in three infants (5.8%) because of respiratory instability. Increased respiratory support within 24 h after the MRI was necessary for 12 infants (23.1%) and was significantly associated with GA, birth weight and the mode of respiratory support. Hypothermia (core temperature < 36°C) occurred in nine infants (17.3%). Temperature dropped significantly after the MRI scan.

**Conclusion:**

Minor adverse events after MRI procedures at 30 weeks GA were common and should not be underestimated. A dedicated and comprehensive guideline for MRI procedures in preterm infants is essential.

**Electronic supplementary material:**

The online version of this article (doi:10.1007/s00247-012-2426-y) contains supplementary material, which is available to authorized users.

## Introduction

In preterm infants, early recognition of neonatal brain injury and assessment of risks of later impairment is a challenging goal of current neuroimaging studies [1–3]. Magnetic resonance imaging (MRI) provides clinicians and researchers objective, high-quality, in vivo information about brain anatomy, pathology and, due to recent advances, functional and physiological characteristics [4–10]. Early cerebral MRI scans at 30 weeks’ postmenstrual age and at term-equivalent age are increasingly being incorporated into standard care for very low birth weight (VLBW) infants (birth weight < 1,500 g). This provides early biomarkers for studying preterm brain injury related to neurodevelopmental outcome. These early determinants may contribute to the design of pharmacological and behavioural interventions to improve outcome [6, 7, 11, 12].

MRI is considered a safe imaging technique. No evidence exists of serious harm to human tissue, besides loud acoustic noise, tissue heating and peripheral nerve stimulation [13–16]. Performing early MRI scans in VLBW infants is challenging, as they frequently require respiratory support and are vulnerable to haemodynamic instability. Consequently, early MRI scans of VLBW infants should be performed in a safe and controlled environment with the use of a dedicated protocol. Studies on the methods that promote patient safety and health care quality are ongoing. Previous studies regarding safety of MRI in VLBW infants suggest that MRI procedures are safe [17–19]. However, population size and maturity range varied widely in these studies, and in some works, only adverse events during the scan were assessed [17, 18].

Our aim was to study the safety of routine MRI scans in preterm infants at a postmenstrual age of 30 weeks. To accomplish this, we collected data of these infants regarding safety incidents and (avoidable) adverse events over a long time period: 24 h before and 24 h following the MRI scan.

## Materials and methods

### Description of the guideline

**Fig. 1:**
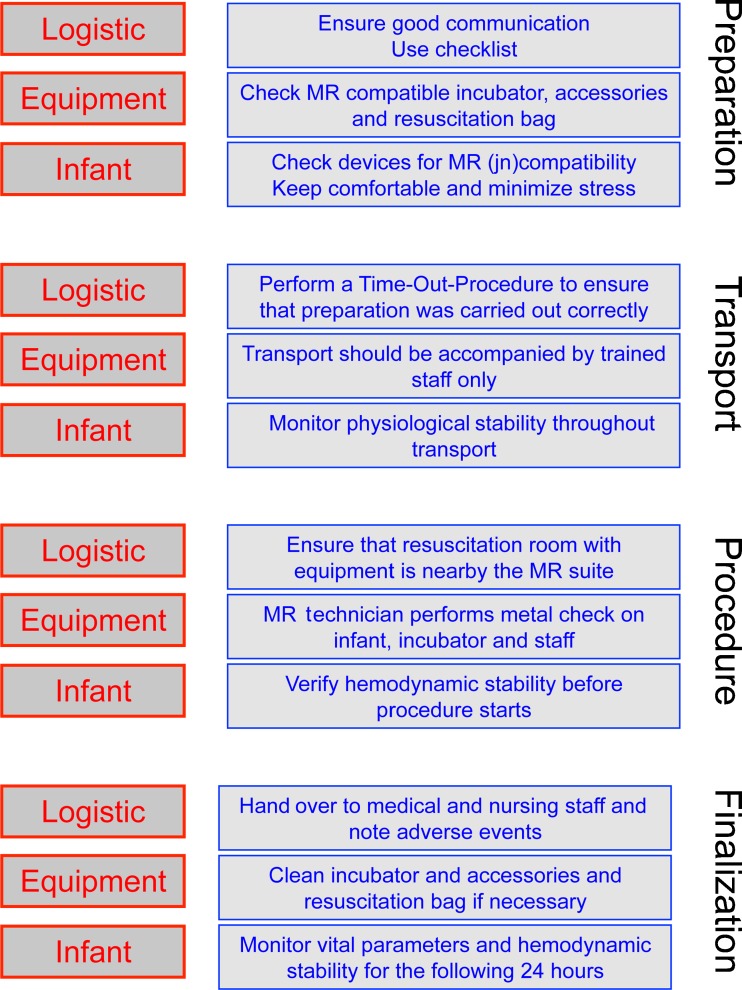
Guideline for safe execution of MRI procedures in preterm infants (see Online Resource 1)

A tailored, centre-specific guideline for MRI procedures in VLBW infants was developed in collaboration with representatives from the radiology and neonatology departments as well as a patient safety officer from the Erasmus Medical Centre, Sophia’s children’s hospital. The guideline was based on the MR safety literature and our own experiences and was adjusted using the principles of the ‘‘plan-do-study-act’’ quality improvement [20], an iterative process, to improve outcomes (Fig. 1). A description of this tailored guideline for MRI procedures in preterm infants is given in Online Resource 1.

### Study participants

As part of standard clinical care practices, MRI scans were performed on VLBW infants that were born at a gestational age (GA) < 29 weeks. These scans were performed at a postmenstrual age of 30 weeks (29 4/7 to 30 4/7 weeks). In all patients, the MRI procedure was carried out according to our multi-disciplinary guideline (see Online Resource 1). The medical team (attending neonatologist and nursing staff) decided whether the infants were medically stable enough to undergo an MRI scan. The following criteria to define haemodynamic and respiratory instability were: high-frequency oscillation (HFO) ventilation, doxapram dependency, inotropic support and sepsis workup within 12 h before the MRI scan.

Data regarding patient safety, such as vital parameters, mode of respiratory support, number of episodes of bradycardia, apnea or oxygen desaturation and (avoidable) adverse events, were retrospectively collected from our electronic patient data management system. These data were sampled at fixed times: 24, 16 and 8 h before the MRI scan, during the MRI procedure itself and 8, 16 and 24 h after the MRI scan. The definitions of major and minor adverse events are listed in Table 1. Increased haemodynamic instability was defined as an increase of more than five episodes of bradycardia (heart rate < 100/min), apnea (> 20 s) or oxygen desaturation (saturation < 85%) within the first 24 h after the MRI compared with the 24 h before the scan. Increased respiratory support within 24 h after the MRI was defined as increased inspiratory pressure, increased positive end expiratory pressure or increased frequency of ventilation. Hypothermia was defined as core temperature < 36°C.

**Table 1:** Definitions of adverse events

Major adverse events	Respiratory compromise resulting in intubation
	Circulatory compromise resulting in need for inotropic agents
	Cardiac resuscitation
	Death
Minor adverse events	Respiratory instability during the procedure
	Respiratory compromise resulting in minor increased respiratory support
	Increased haemodynamic instability
	Hypothermia

The study was approved by the medical ethical committee of the Erasmus Medical Centre Rotterdam, The Netherlands.

### Image acquisition

All MRI scans were performed using a 1.5-T GE Echo Speed scanner (GE Healthcare, Milwaukee, WI, USA). The standard imaging protocol included the following: axial T1-weighted spin echo, axial T2-weighted dual spin echo, sagittal T1-weighted spin echo, axial 3-D T1-weighted SPGR and echo planar diffusion tensor imaging. The acquisition times were approximately 5-6 min per sequence.

### Statistical analysis

Statistical analysis was performed using SPSS v17.0.2 (SPSS, IL, USA). A repeated measures ANOVA using Wilks lambda test was conducted to test the stability of vital parameters during the MRI procedure. Correlations of adverse events with GA, birth weight, weight at image acquisition, gender, temperature drop, mode of respiratory support and total acquisition time were tested. Pearson correlation coefficients were used for continuous variables. Pearson chi-squared test was performed for proportional differences between two categorically scaled variables. One-way ANOVA was used for mean differences among three or more groups holding the groups as a factor variable categorically scaled. A *P* value of < 0.05 (two-sided) was considered statistically significant.

## Results

A total of 158 infants were eligible for inclusion in the study. Among these, 32 infants died before term-equivalent 30 weeks’ GA, 36 infants were transferred to other hospitals before the MRI scan could be performed and in 38 infants the MRI scan was postponed because the infants were not haemodynamically stable enough for MRI scanning at 30 weeks’ GA. Therefore, 52 infants (30 boys) underwent a cerebral MRI scan. Patient characteristics are listed in Table 2.

**Table 2:** Patient characteristics

Gestational age at birth, mean ± SD [weeks]	26.8 ± 1.4
Birth weight, mean ± SD [g]	967 ± 247
Postmenstrual age at MR acquisition, mean ± SD [weeks]	30.1 ± 0.3
Weight at MR acquisition, mean ± SD [g]	1,133 ± 197
Male gender *n* (%)	30 (57.7)
Mode of respiratory support during MRI [*n* (%)]	
Mild respiratory support [*n* (%)]	
Nasal prongs	10 (19.2)
CPAP	27 (51.9)
Moderate respiratory support [*n* (%)]	
Non-invasive ventilation	11 (21.2)
Mechanical ventilation	4 (7.7)
Total acquisition time, mean ± SD [min]	39 ± 13

### Adverse events

**Fig. 2:**
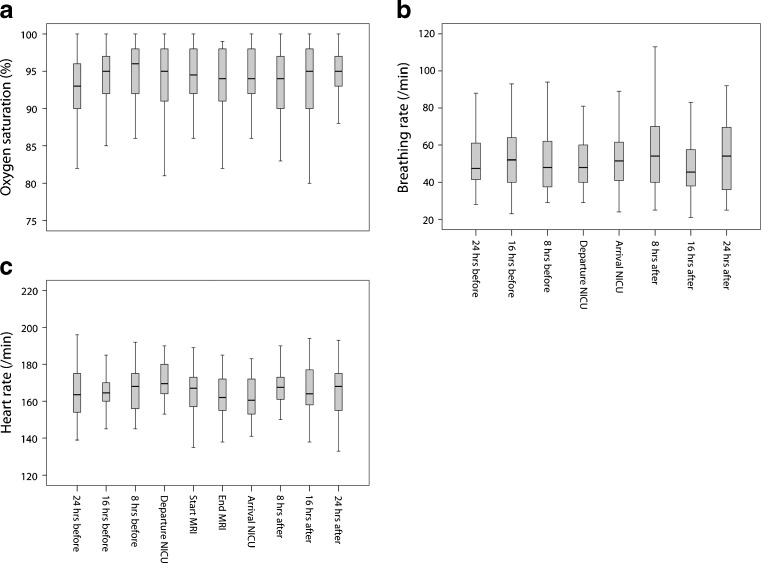
Vital parameters during the MRI procedure. Trend of oxygen saturation (**a**), breathing rate (breathing rate was not measured during the MRI scan) (**b**) and heart rate (**c**) during the 48 h surrounding the MRI scan. Note that, generally, these parameters remained stable

Generally, compared with 24 h before the MRI scan, vital parameters (heart rate, breathing rate and oxygen saturation) remained stable during the 24 h after the scan (Fig. 2). Increased haemodynamic instability occurred in 14 infants (26.9%) (Table 3).

**Table 3:** Minor adverse events related to MRI procedure

	*n* (%)
Increased haemodynamic instability	14 (26.9)
Cancellation of MRI because of respiratory instability	3 (5.8)
Obstruction of central venous catheter after MRI	1 (1.9)
Increased respiratory support necessary within 24 h after MRI	12 (23.1)
Hypothermia (< 36.0˚) after MRI	9 (17.3)
Total number of adverse events	39

Even though vital parameters remained stable during the MRI scan itself, increased haemodynamic instability occurred within the following 24 h in some infants.

**Fig. 3:**
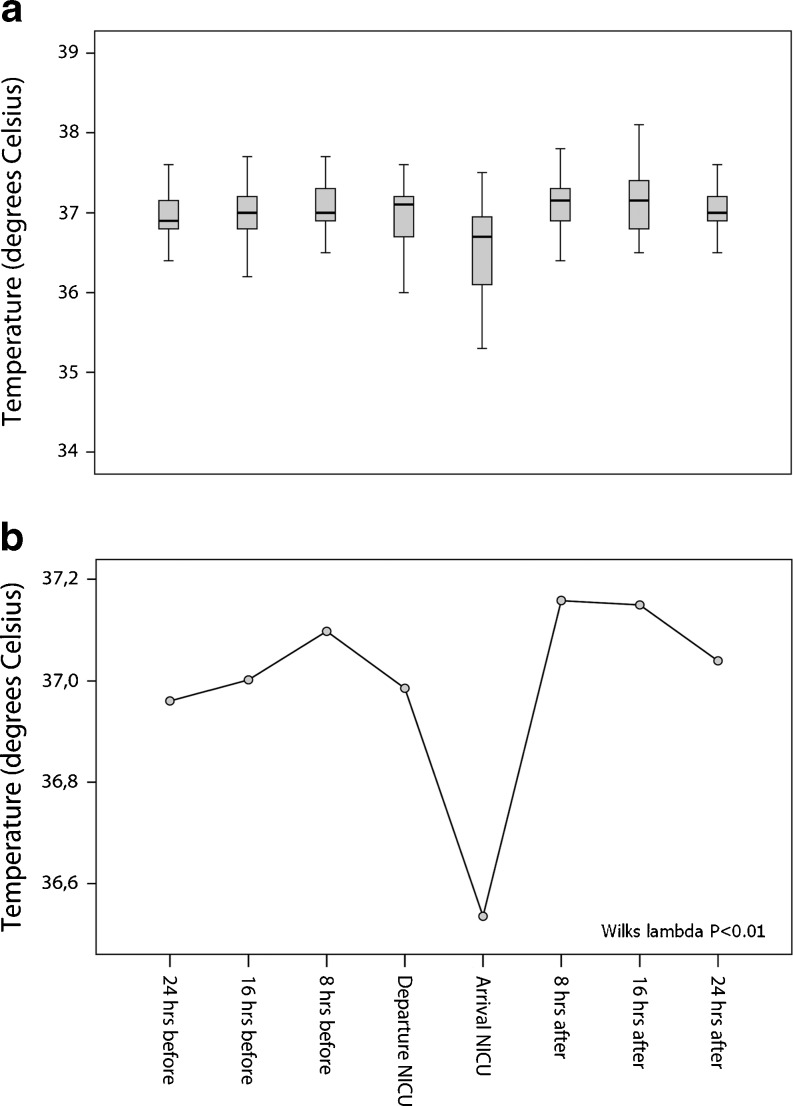
Temperature measurements during the MRI procedure. Trend of temperature during the 48 h surrounding the MRI scan (**a**). Repeated measures ANOVA shows that temperature dropped significantly after the MRI scan (**b**)

No adverse events occurred in 26 infants (50%). However, in 26 infants (preventable) incidents and minor adverse events were encountered (Table 3). The MRI scan was cancelled in three infants (5.8%) because of respiratory instability. In another infant, obstruction of the central venous catheter occurred after the scan, although its cause is unclear. Twelve infants (23.1%) needed increased respiratory support within 24 h after the MRI. In one infant, this might have been due to being transported twice to the MR scanning room because of technical problems with the magnet. Only two infants needed an increased mode of respiratory support: from continuous positive airway pressure (CPAP) to non-invasive ventilation. Infants that required increased respiratory support after the scan were born at a significantly lower GA, were born with a lower birth weight and/or more frequently received moderate respiratory support during the scan (Table 4). Hypothermia occurred in nine infants (17.3%). On average, the infants’ core temperature dropped 0.5 degrees after the MRI scan. Temperature was significantly decreased after MRI scanning (Fig. 3).

**Table 4:** Comparisons of increased respiratory support with other variables

Parameter		Need for increased respiratory support	*P*
		No	Yes
Gestational age at birth, mean ± SD [weeks]	27.1 ± 1.3	25.8 ± 1.4	< 0.01^a^
Birth weight, mean ± SD [g]	1007 ± 244	831 ± 210	0.03^a^
Weight at MR acquisition, mean ± SD [g]	1146 ± 210	1091 ± 148	NS^a^
Temperature drop after MRI procedure [°C]	0.5 ± 0.6	0.5 ± 0.5	NS^a^
Male gender [*n*]	21	9	NS^b^
Mode of respiratory support during MRI [*n*]			0.03^b^
Mild respiratory support	32	5	
Moderate respiratory support	8	7	

*NS* not significant

^a^Pearson’s *T*-test

^b^Fisher’s exact test

## Discussion

Our study stresses the importance of providing a controlled environment for early MRI procedures for preterm infants. Despite the presence of a multi-disciplinary guideline specifically designed for preterm infants, minor adverse events, such as hypothermia and the need for increased respiratory support after the scan, were encountered regularly: these events occurred in 26 infants, 50% of our study population. In total, 39 minor adverse events occurred. Therefore, caution needs to be taken regarding the safety of VLBW infants during MRI procedures. Critical incident review and continuous re-evaluation of the guidelines are essential in this process.

MRI is becoming increasingly important for accurately evaluating brain injuries and the consequent effects on neurodevelopment in preterm infants [9, 11, 21, 22]. Compared with cranial US, MRI has proven to be more sensitive for the detection of diffuse white matter injury (DWMI) [3, 23, 24], and allows objective quantification of brain injury at a micro-structural level [4, 25]. MRI is considered a safe imaging technique, independent of ionizing radiation, and it enables high-resolution neuroimaging in a non-invasive manner [26]. The use of MRI scanning is limited in preterm infants because of their cardio-respiratory instability and predisposition to hypothermia [17, 26–28]. Performing an MRI scan in this vulnerable population requires a comprehensive guideline that includes all the essential elements: good preparation, optimal monitoring of vital parameters, open communication between the involved parties, individualised care and continuous efforts to improve the quality of care. Neonatal intensive care must obviously be maintained throughout the procedure, which requires the use of MR-compatible equipment that ensures optimal monitoring of vital parameters without causing injuries, such as burning, or image degradation as a result of radiofrequency interference with the static magnetic field.

Because of the increased risk of respiratory and circulatory compromise, no sedation was used in this study. To reduce motion artefacts, we use other strategies to comfort the infant, such as those according to the principles of the Newborn Individualised Developmental Care and Assessment Program [29, 30].

Safety incidents in (neonatal) health care are generally related to poor preparation, equipment failure and human error [31, 32]. Studies on interventions to improve healthcare quality, such as staff training, implementation of a time-out-procedure (TOP) and the use of checklists and tailored guidelines, have shown that such preventable incidents can be reduced [33–35]. In addition, adverse events should always be reported in order for the guideline to be adjusted [31]. Comparable to operative procedures, a systematic pre-procedural briefing, such as a TOP, can be implemented for MR procedures as well. A pre-procedural TOP ensures that all involved caregivers agree that the correct procedure is being carried out properly for the correct patient.

We have shown that adverse events related to MRI scans in this vulnerable population are common. This is in contrast to other studies [17–19], in which no significant adverse events were found. However, these studies primarily investigated serious adverse events that occurred during the scan itself, and the MRI scans had short acquisition times [17], or the study population consisted of patients with a wide range of gestational ages [17, 18]. In contrast, the results of the current study only include data on VLBW infants with a mean postmenstrual age of 30 weeks ± 4 days. In addition, we collected data for the 48 h surrounding the MRI procedure and total acquisition time was approximately 39 min.

Although it is reasonable and logical to assume that a longer total acquisition time is likely to increase the number of adverse events, we were unable to demonstrate this relationship in our study, possibly related to the small sample size.

The limitations of this study include selection bias, as our data consist only of infants considered haemodynamically stable enough for an MRI scan. In our setting, the medical team decided whether the infants were haemodynamically stable enough to undergo an MRI scan.

Perhaps if more strict criteria for haemodynamic stability were applied, the incidence of adverse events might be less frequent. In contrast, the incidence of adverse events might increase if more critically ill preterm infants were scanned, emphasising the importance of a comprehensive guideline with strict contraindications and staff training to ensure the safe execution of MR procedures.

Another limitation could be the retrospective design and the lack of temperature measurement during the MRI scan. Despite the use of an MR-compatible incubator, which provides controlled temperature and humidity, we encountered an increased incidence of hypothermia after the MRI scan. This could be explained by the mode of respiratory support: the infants were supported with cold air or oxygen during the procedure, which is in contrast to the setting at our wards, where infants are supported with pre-heated (40°C) air or oxygen. In order to decrease the high incidence of hypothermia after the MRI scan, we propose using an optical temperature probe to measure temperature continuously during the scan. Although minor adverse events were encountered more frequently after the MRI scan, it is not with certainty established that this in fact can be attributed to having undergone an MRI scan. Being transported from the NICU alone could in fact be stressful enough. However, due to the lack of evidence against causality and in the context of patient safety, we argue that each adverse event should be considered as a result of the procedure. Moreover, in order to avoid this possible bias, vital parameters, mode of respiratory support and the number of episodes of bradycardia, apnea or oxygen desaturation that occurred within 24 h before the MRI scan were compared with the same details occurring within 24 h following the MRI scan of each infant individually.

Logistical regression to weigh gestational age, birth weight and mode of respiratory support with the increased need for respiratory support was not performed given the small sample size (*n* = 12) in that group.

Finally, no serious adverse events occurred during the procedures, but the clinical significance of minor adverse events for future neurodevelopmental outcome remains unclear. Until empirical evidence shows that these events do not adversely affect neurodevelopment, we argue that adverse events should always be considered potentially harmful, and maximal efforts to prevent them must be undertaken.

## Conclusion

Adverse events within 24 h after routine MRI procedures in VLBW infants at 30 weeks’ gestational age are common, 50% of the MRI procedures in this study were complicated by a minor adverse event. Our findings illustrate the importance of providing a safe environment for early MRI procedures in preterm infants. Considering the increased application of MRI as part of the standard clinical care for preterm infants, a multi-disciplinary-based approach with continuous re-evaluation of the guidelines is necessary to ensure optimal safety for this population.

## Electronic supplementary material

Below is the link to the electronic supplementary material.

ESM 1 (DOC 30 kb)
