# Supplementary material for: Safety of routine early MRI in preterm infants
Source: Pediatr Radiol. 2012 Aug 9;42(10):1205–11. doi: 10.1007/s00247-012-2426-y (PMC3460174; doi:10.1007/s00247-012-2426-y)
Supplement: Supplementary file 1 — (DOC 30 kb) [file 247_2012_2426_MOESM1_ESM.doc]

**Online Resource 1: tailored guideline for MRI procedures in preterm infants**

**‘Safety of routine early MRI in preterm infants’ – Pediatric Radiology**

**Preparation**
The medical team (attending neonatologist, pediatric radiologist and nursing staff) decides whether the infant is medically stable enough to undergo an MRI scan and whether the scan is indicated.

A multidisciplinary approach with close communication is essential.

A checklist is used to prepare the infant and equipment for the procedure; this checklist ensures a minimal risk of adverse events related to incorrect execution of the procedure.

An MR-compatible incubator is used, which provides controlled temperature and humidity as well as MR-compatible pulse oximetry and ventilation. The MR-compatible incubator is checked as follows: the temperature is set as it is set on the infant’s own incubator; nonmagnetic air and oxygen tanks are present with sufficient capacity; and equipment for ventilation support is available and working.

A resuscitation bag with all necessary equipment for acute interventions is checked and available during the procedure.

All devices attached to the infant and implants (e.g., ECG leads, pulse oximetry probe, temperature probe, intravascular catheters, ductus arteriosus clips and ventriculo-peritoneal shunts as well as metal-containing infant clothing and bracelets) are checked for MR compatibility (http://www.mrisafety.com). MR-compatible ECG electrodes and pulse oximetry probe are attached to the infant to monitor heart rate and oxygen saturation during the scan.

The infant is protected against noise with moldable earplugs and neonatal earmuffs (Minimufs, provide around 7 Db attenuation).

Infusion lines are sufficiently extended such that the infant can undergo an MRI scan while the infusion pumps remain outside the scanning room, or MR-compatible infusion pumps can be used.

The infant is placed in the MR incubator in a comfortable and secure way with small cushions to encourage sleep and reduce movement. As sedation can cause respiratory and circulatory compromise, we do not use sedation in preterm infants.

**Transport**

A time-out procedure is performed before leaving the NICU such that a quick re-check is conducted and all involved parties agree on the following: the correct infant has been properly prepared, the MR incubator is set correctly, the infant is stable and comfortable and the MR department is ready to scan the infant.

Transport is accompanied by trained staff, and physiological stability is monitored during transport.

**During the acquisition**

Staff trained in neonatal life support remain present throughout the MRI scan.

A room near the MR suite with equipment, supplies and guidelines for neonatal resuscitation is checked and available during the MRI scan.

The technician at the MR suite performs a metal check on the infant, incubator, oxygen and air tanks and accompanying staff before entering the MR suite. Because of the potential hazards associated with the strong electromagnetic field, MR safety training for all accompanying staff is recommended and provided in our setting.

Before the actual MR procedure starts, the presence of adequate respiratory support, hemodynamic stability and the infant’s comfort are verified.

Hemodynamic stability is closely monitored from the MRI incubator’s screen, which can be seen from the console room. The MRI procedure should be interrupted if hemodynamic stability is compromised or if there is any doubt about it.

**After the MRI scan**

After the acquisition, the infant’s hemodynamic stability and respiratory support are checked again before returning to the NICU.

Upon arrival to the NICU, a handover of the procedure to medical and nursing staff takes place, and possible adverse events are noted.

The MR-compatible incubator and accessories are cleaned, and the resuscitation bag is refilled if necessary.

During the subsequent 24 hours, the infant’s vital parameters and hemodynamic stability are monitored continuously.
